# Germline Mutations in the Polyposis-Associated Genes *BMPR1A*, *SMAD4, PTEN, MUTYH* and *GREM1* Are Not Common in Individuals with Serrated Polyposis Syndrome

**DOI:** 10.1371/journal.pone.0066705

**Published:** 2013-06-21

**Authors:** Mark Clendenning, Joanne P. Young, Michael D. Walsh, Sonja Woodall, Julie Arnold, Mark Jenkins, Aung Ko Win, John L. Hopper, Kevin Sweet, Steven Gallinger, Christophe Rosty, Susan Parry, Daniel D. Buchanan

**Affiliations:** 1 Cancer and Population Studies Group, Queensland Institute of Medical Research, Brisbane, Queensland, Australia; 2 Department of Histopathology, Sullivan Nicolaides Pathology, Brisbane, Queensland, Australia; 3 New Zealand Familial Gastrointestinal Cancer Service, Auckland Hospital, Auckland, New Zealand; 4 Centre for MEGA Epidemiology, University of Melbourne, Melbourne, Victoria, Australia; 5 Division of Human Genetics, The Ohio State University Medical Centre, Columbus, Ohio, United States of America; 6 Cancer Care Ontario, Toronto, Ontario, Canada; 7 Samuel Lunenfeld Research Institute, Mount Sinai Hospital, Toronto, Ontario, Canada; 8 Zane Cohen Centre for Digestive Diseases, Mount Sinai Hospital, Toronto, Ontario, Canada; 9 Department of Molecular and Cellular Pathology, University of Queensland, Brisbane, Queensland, Australia; 10 Envoi Specialist Pathologists, Brisbane, Queensland, Australia; 11 Department of Gastroenterology and Hepatology, Middlemore Hospital, Auckland, New Zealand; Ohio State University Medical Center, United States of America

## Abstract

**Background:**

Recent reports have observed that individuals with serrated polyps, some of whom meet the clinical diagnostic criteria for Serrated Polyposis Syndrome (SPS), are among those who carry germline mutations in genes associated with polyposis syndromes including; (1) genes known to underlie hamartomatous polyposes (*SMAD4*, *BMPR1A*, and *PTEN*), (2) *MUTYH*-associated polyposis and (3) *GREM1* in Hereditary Mixed Polyposis Syndrome (HMPS). The aim of this study was to characterise individuals fulfilling the current WHO criteria for SPS for germline mutations in these polyposis-associated genes.

**Methods:**

A total of 65 individuals with SPS (fulfilling WHO criteria 1 or 3), were recruited to the Genetics of Serrated Neoplasia study between 2000 and 2012, through multiple Genetics or Family Cancer Clinics within Australia, or from the New Zealand Familial Gastrointestinal Cancer Service. Individuals with SPS were tested for coding mutations and large deletions in the *PTEN*, *SMAD4,* and *BMPR1A* genes, for the *MUTYH* variants in exons 7 (Y179C) and 13 (G396D), and for the duplication upstream of *GREM1*.

**Results:**

We found no variants that were likely to be deleterious germline mutations in the SPS cases in the *PTEN*, *SMAD4,* and *BMPR1A* genes. A novel variant in intron 2 (c.164+223T>C) of *PTEN* was identified in one individual and was predicted by *in silico* analysis to have no functional consequences. One further individual with SPS was found to be mono-allelic for the *MUTYH* G396D mutation. No individuals carried the recently reported duplication within *GREM1*.

**Conclusions:**

Genes involved in the gastrointestinal hamartomatous polyposis, Hereditary Mixed Polyposis Syndrome and *MUTYH*-associated polyposis syndromes are not commonly altered in individuals with SPS.

## Introduction

Serrated Polyposis Syndrome (SPS), previously known as hyperplastic polyposis syndrome, is a colorectal polyposis condition of unknown genetic basis associated with an increased risk of developing colorectal cancer (CRC) in both the affected individual and their first- and second-degree relatives [1]. CRC has been detected in 25–50% of individuals with SPS, most often identified at time of cancer diagnosis [2], [3], [4], [5], [6], [7], [8]. SPS was first described in the 1970s [9], however, it has only recently been recognised as a condition with a potential genetic basis, with a reported five-fold increase in risk of CRC [1], [10], and a 3.5-fold increase in risk of pancreatic cancer for first-degree relatives of individuals with SPS [1]. SPS is characterised by the occurrence of multiple serrated polyps in the colon and/or rectum, including hyperplastic polyps and sessile serrated adenomas. In addition, conventional adenomas of the large intestine are also identified in up to 80% of individuals with SPS [6], [11], [12] and have been found to be more frequently present in CRC-affected individuals with SPS. SPS is currently diagnosed by the following criteria as defined by the WHO in 2010 [13]:

1. at least 5 serrated polyps proximal to the sigmoid colon, with 2 or more of these being >10 mm; *OR*
2. any number of serrated polyps proximal to the sigmoid colon in an individual who has a first-degree relative with serrated polyposis; *OR*
3. >20 serrated polyps of any size but distributed throughout the colon.

Other rare colonic polyposis syndromes for which the genetic basis is known include Cowden syndrome (mutations in *PTEN*), juvenile polyposis (mutations in *SMAD4* or *BMPR1A*), hereditary mixed polyposis syndrome (mutation in *GREM1*), and *MUTYH*-associated polyposis (biallelic mutations in *MUTYH*). Cowden syndrome is part of a disease complex known as the PTEN hamartoma tumour syndrome (PHTS) and is associated with macrocephaly, malignant and benign tumours of the endometrium, thyroid and breast [14], and hamartomatous polyposis of the gastrointestinal tract [15]. Other polyp sub-types have also been reported in individuals with germline *PTEN* mutations. In a recent article by Heald *et al.* [16], 40% of the individuals who underwent colonoscopy were found to have serrated (hyperplastic) polyps. Among those individuals with serrated polyps, 60% met the current WHO clinical criteria for SPS [13].

Multiple serrated polyps and adenomas have also been described in individuals with *BMPR1A* [17], biallelic *MUTYH* mutations [18] and *SMAD4* mutation [19], genes that are normally associated with juvenile or adenomatous polyposes. In addition, mutations in *GREM1*, which plays a role in the inhibition of BMP signalling, have been reported to underlie Hereditary Mixed Polyposis Syndrome (HMPS), a rare condition characterised by the occurrence of conventional adenomas, atypical hamartomatous polyps, serrated polyps and frequent colorectal carcinoma [20], [21]. Given SPS is observed in mutation carriers associated with these polyposes syndromes, we hypothesised that these genes may be altered in individuals with SPS and may account, at least partially, for the development of this condition. The aim of this study was to determine the frequency of germline mutations in the *PTEN*, *SMAD4, BMPR1A, MUTYH* and *GREM1* genes in a large series of individuals with SPS.

## Methods

### Ethics Statement

Written informed consent was obtained from all study participants and the study protocol was approved by the Human Research Ethics Committee of the Queensland Institute of Medical Research under protocol P912.

### Study Participants

A total of 65 individuals with SPS, meeting WHO criteria 1 or 3 [13] and with DNA available for genetic testing, recruited from multiple Genetics or Family Cancer Clinics within Australia (AUS), or from the New Zealand Familial Gastrointestinal Cancer Service, Auckland, New Zealand (NZL), between 2000 and 2012 to the Genetics of Serrated Neoplasia (GSN) study [1], [11], [12] were selected for this study. All included individuals with SPS were recruited regardless of a family history of polyps or cancer. All SPS cases in this study were Caucasian. Individuals who fulfilled WHO criteria 2 [13] were not included in this study of unrelated SPS cases as, by definition, they were likely to be relatives of the 65 SPS probands.

### Gene Testing

Individuals with SPS were screened for mutations in the coding regions and directly flanking introns in the *PTEN*, *SMAD4* and *BMPR1A* genes as follows. Standard PCR was performed to amplify 50 ng of buffy coat derived DNA across exons 1–9 of *PTEN* (NM_000314.4), exons 2–12 of *SMAD4* (NM_005359.5), and exons 3–13 of *BMPR1A* (NM_004329.2) using GoTaq mastermix (Promega, Madison, WI), (primer sequences available on request). PCR products underwent clean-up using Multi-screen HTS PCR plates (Millipore, Billerica, MA). Sequencing was performed in a single direction using ABI BigDye v3.1, and subsequently treated with ABI Xterminator reagent (Life Technologies, Carlsbad, CA). Sequencing reactions were run on an ABI 3100 genetic analyser, and annotated using DNA Star Lasergene 8 software (DNAstar, Madison, WI). To identify large rearrangements (deletions or duplications) in *PTEN*, *SMAD4* and *BMPR1A*, multiplex ligation-dependent probe amplification (MLPA) was performed using the JPS MLPA kit (P158; MRC Holland).

The common European variants of *MUTYH,* c.536A>G p.Tyr179Cys (Y179C) in exon 7 and c.1187G>A p.Gly396Asp (G396D) in exon 13, were tested using a high resolution melt curve (HRM) analysis assay. Briefly, 10 ng of DNA was amplified in a 15 µl reaction, containing 300 nM of each primer (1F-5′-TCCTACCCACAGGAGGTGAA-3′ and 2R-5′-CCTGCCATCCCCTTACCTT-3′ for the Y197C variant or 1F-5′- GGGCAGTGGCATGAGTAAC-3′ and 2R-5′- GACGGGAACTCCCACAGTC-3′ for the G396D variant), 1U of platinum Taq, 1.5 mM of MgCl_2_ (Life Technologies, Carlsbad, CA), 200 nM of dNTPs and 1 µM of SYTO9 dye (Life Technologies, Carlsbad, CA), under PCR cycling conditions of denaturation at 95°C for 2 mins followed by 40 cycles of 95°C for 15 secs, 62°C (Y179C) or 66°C (G396D) for 15 secs and 72°C for 15 secs. A final high resolution melting analysis was performed by increasing 0.25°C temperature steps from 80°C until complete denaturation at 95°C. Melting curves for individual samples were compared to reference samples for each of the three genotypes for either the Y179C or G396D variants that had been previously confirmed by Sanger sequencing.

A heterozygous single-copy duplication of approximately 40 kb on chromosome 15q13.3 (30,752,231–30,792,051) was recently identified in individuals with HMPS [20]. The duplication resulted in an insertion of a 30-bp sequence of unknown origin and extended from intron 2 of *SCG5* to a site just upstream of the *GREM1* CpG island. A PCR product spanning the duplication boundary produced a unique 190 bp product in carriers with a separate PCR product of 435 bp upstream of *GREM1* used as a control sequence for amplification. Products were amplified in a 15 ul reaction using GoTaq mastermix (Promega, Madison, WI) and 300 nM of each primer 1F-5′-GGGCATCTTCTGGTCTCT-3′ and 2R-5′- AGTGAGACCTGGGGAAAG-3′ for the 190 bp duplication product or 1F-5′- GGGCATCTTCTGGTCTCT-3′ and 2R-5′- CGACCGGGTCTTATGTATC-3′ for the 435 bp control product. PCR cycling conditions included a denaturation step at 95°C for 2 mins followed by 40 cycles of 95°C for 15 secs, 60°C for 15 secs and 72°C for 15 secs. All products were separated on a 2% agarose gel alongside a positive control for the duplication.

## Results

The baseline characteristics of the 65 individuals with SPS are shown in Table 1. Briefly, the average age at diagnosis of SPS was 51.5 yrs with standard deviation (SD) of 15.3 yrs with a female predominance (60%). The average polyp count was 31.7 (SD = 23.5) with 83.1% of the individuals with SPS having >20 serrated polyps of any size throughout the colon (fulfilling WHO criteria 3). A personal history of CRC or having a first-degree relative with CRC was reported in 32.3% and 23%, respectively.

**Table 1:** Characteristics of SPS cases in the study.

		N (%)	
Sex	female	39 (60%)	
	male	26 (40%)	
Age at Diagnosis (yrs)	mean	51.5	
	SD	15.3	
	min	18	
	max	79	
Polyp count (min)*	mean	31.7	
	SD	23.5	
	min	5	
	max	130	
WHO criteria≠	1	6 (9.2%)	
	3	54 (83.1%)	
	Inconclusive∧	5 (7.7%)	
Developed CRC	No	44 (67.7%)	
	Yes	21 (32.3%)	
First-degree relative with CRC	No	37 (56.9%)	
	Yes	28 (43.1%)	

* minimum number of polyps observed, polyp count may be a cumulative count.

≠ The frequency of individuals with SPS that meet WHO criteria 1 or 3, where individuals that met WHO criteria 2 only weren’t included.

∧ WHO criteria could not be determined as polyp count listed as “multiple” but >5.

No deleterious germline mutations in *PTEN* were identified in the 65 individuals with SPS. The c.80–96A>G (rs1903858) polymorphism was identified in one individual. In a single SPS individual, we also identified a novel variant in intron 2 (c.164+223T>C) of *PTEN*, which was predicted by *in silico* analysis (NNSPLICE 0.9 at http://www.fruitfly.org/seq_tools/splice.html) to have no functional consequences. In addition, no deleterious germline mutations in *BMPR1A* or *SMAD4* were identified in the 65 SPS individuals. Similarly, no large deletions or duplications in *PTEN, BMPR1A* or *SMAD4* were observed.

We found no biallelic mutation carriers for the *MUTYH* variants c.536A>G p.Tyr179Cys (Y179C) in exon 7 and c.1187G>A p.Gly396Asp (G396D) in exon 13, after screening with a novel high resolution melt curve (HRM) analysis assay. However, a single SPS case was shown to be a monoallelic carrier of the G396D variant. The recently described duplication upstream of *GREM1* was screened for in all sixty-five SPS individuals with no carriers being identified.

## Discussion

SPS is a clinically defined condition by arbitrary criteria for which a genetic cause is yet to be identified. The observation that multiple colonic serrated polyps, including in individuals meeting the clinical criteria for SPS, has been observed among mutations carriers of genes causing other rare polyposes syndromes (*PTEN* [*16*], *BMPR1A* [*17*], *MUTYH* [18], *SMAD4* and *GREM1*) raises the question that these genes should be tested for in individuals with SPS. In this study of 65 individuals with well characterised SPS, we did not observe any deleterious mutations in the *PTEN*, *BMPR1A* or *SMAD4* genes or any biallelic carriers of the most common *MUTYH* mutations, Y179C and G396D, and no evidence of the duplication upstream of the *GREM1* gene. Although we did not identify mutations within these genes, other transcriptional silencing or protein disrupting mechanisms such as intronic mutations or germline gene promoter methylation (epimutations), as can occur in the mismatch repair genes [22], [23], cannot be excluded.

The polyp burden for individuals with Cowden syndrome is relatively high, with greater than 50 polyps present in over half of these individuals [24]. Though the majority of polyps observed in individuals with germline mutations in *PTEN* are hamartomas, ganglioneuromas and inflammatory polyps, one recent study observed an unexpectedly high incidence of individuals (24%) with co-existent serrated polyps that met the clinical criteria for SPS [16]. A previous report from Sweet *et al.* [25] found two *PTEN* mutation carriers within 23 individuals with co-existent SPS (9%). In the report by Heald *et al.* [16] the authors state that the majority of individuals with *PTEN* mutation had macrocephaly, as did both individuals with SPS reported by Sweet *et al.* [25]. In contrast, we observed no *PTEN* mutations in our 65 individuals with SPS. Therefore, in the absence of any other Cowden-like phenotypic features, macrocephaly could potentially be used to guide *PTEN* mutation screening in individuals presenting with SPS.

Multiple serrated polyps are also seen in *MUTYH*-associated polyposis, a disorder with a variety of phenotypes ranging from no gastrointestinal polyps through adenomatous polyps to mixed polyposis phenotypes [26]. In one study, 18% of biallelic mutation carriers met the clinical criteria for SPS [18], further supporting the concept of phenotypic variability due to differing genetic backgrounds. We have previously shown that, similarly to *PTEN*, although SPS is observed among *MUTYH* mutation carriers, *MUTYH* mutations are rare amongst individuals with SPS [27]. Therefore, the observations from these previous studies and the current study (only a single monoallelic *MUTYH* mutation carrier identified in 65 SPS individuals) further support the concept that the common *MUTYH* mutations in Europeans do not underlie the majority of SPS. However, this does not exclude the possibility that other variants within the *MUTYH* gene may be associated with risk of SPS.

In addition, individuals with germline mutations in the *BMPR1A* and *SMAD4* genes may also express variable phenotypes, some of which include multiple serrated polyps of the large intestine. Case reports have shown that in *BMPR1A* families with polyposis phenotypes, polyps of both serrated and adenomatous lineages are present [28], Juvenile polyps dominate the gastrointestinal phenotype in *SMAD4* mutation carriers, however, a deleterious frameshift mutation in *SMAD4* has been described in a case of juvenile polyposis previously classified as SPS, where multiple serrated polyps were also present [19]. Therefore, the possibility exists that germline mutations in these polyposis-associated genes may be interacting with a more common genetic background present in the population to produce a phenotype of SPS in a subset of individuals.

Hereditary Mixed Polyposis Syndrome (HMPS) is characterised by the development of polyposis with mixed polyp morphologies including conventional adenomas, serrated polyps, atypical juvenile polyps, and an increased risk of CRC [29]. The recently reported 40 kb duplication upstream of the *GREM1* gene that results in the increased expression of *GREM1* mRNA has been demonstrated to underlie HMPS, however, the families that have thus far been shown to carry this mutation have been of Ashkenazi Jewish descent [20]. In contrast, current evidence suggests that SPS [6], and serrated polyps in general [30], are significantly more common in northern Europeans of primarily Anglo-Celtic descent, therefore, the absence of the duplication upstream of *GREM1* in SPS cases in this study is not surprising. Furthermore, in this study, we tested only for the 40 kb duplication associated with HMPS and therefore, the possibility exists that other genetic variants within the *GREM1* locus may underlie SPS. Additional evidence to support this region in colonic neoplasia predisposition resides with SNPs within the *GREM1* locus (rs16969681 and rs11632715) that were associated with CRC risk from genome-wide association studies [31] and the description of a whole-gene duplication of *GREM1* described in a single CRC-affected individual [32].

SPS has recently gained recognition as an inferred genetic disorder associated with an increased risk for CRC in both individuals [3], and a five-fold increased risk for CRC in first-degree relatives [1], [10]. It is not known whether SPS individuals reported in the polyposis-associated gene mutation carriers have the same increased risk of CRC. The suggestion of Heald *et al* [*16*] that this phenotype in individuals with *PTEN* mutation be considered as a marker for increased CRC risk is an important one. Though rare reports of SPS in individuals with germline *PTEN* mutation resulting directly from *PTEN* mutation itself cannot be ruled out, it is also possible that such individuals may be segregating more than one disorder. This concept has been previously demonstrated in two kindreds segregating Lynch syndrome and a predisposition for serrated neoplasia [33], and therefore, in families presenting with serrated polyps and with a mutation in one of these polyposis-associated genes, CRC surveillance may be warranted in individual relatives who *do not* carry the family mutation.

In conclusion, we found no germline deleterious mutation in *PTEN*, *BMPR1A*, or *SMAD4* genes, no biallelic carriers of the common *MUTYH* mutations and no evidence of the duplication involving *GREM1*, in our series of 65 well characterised SPS individuals. The observation that mutations in the *PTEN*, *SMAD4*, *BMPR1A, MUTYH* and *GREM1* genes are rarely observed in individuals with SPS may help inform decision making for future genetic screening.
